# Decrypting tubby-like protein gene family of multiple functions in starch root crop cassava

**DOI:** 10.1093/aobpla/plz075

**Published:** 2019-11-25

**Authors:** Ming-You Dong, Xian-Wei Fan, Xiang-Yu Pang, You-Zhi Li

**Affiliations:** State Key Laboratory for Conservation and Utilization of Subtropical Agro-Bioresources, College of Life Science and Technology, Guangxi University, Nanning, Guangxi, China

**Keywords:** Cassava, evolution, gene expression, proteins, growth and development, stress tolerance, tubby-like protein gene family

## Abstract

Tubby-like proteins (TLPs) are ubiquitous in eukaryotes and function in abiotic stress tolerance of some plants. Cassava (*Manihot esculenta* Crantz) is a high-yield starch root crop and has a high tolerance to poor soil conditions and abiotic stress. However, little is known about TLP gene characteristics and their expression in cassava. We identified cassava TLP genes, *MeTLP*s, and further analysed structure, duplication, chromosome localization and collinearity, *cis*-acting elements in the promoter regions and expression patterns of *MeTLP*s, and three-dimensional structure of the encoded proteins MeTLPs. In conclusion, there is a *MeTLP* family containing 13 members, which are grouped into A and C subfamilies. There are 11 pairs of *MeTLP*s that show the duplication which took place between 10.11 and 126.69 million years ago. Two *MeTLP*s *6* and *9* likely originate from one gene in an ancestral species, may be common ancestors for other *MeTLP*s and would most likely not be eligible for ubiquitin-related protein degradation because their corresponding proteins (MeTLPs 6 and 9) have no the F-box domain in the N-terminus. *MeTLP*s feature differences in the number from *TLP*s in wheat, apple, *Arabidopsis*, poplar and maize, and are highlighted by segmental duplication but more importantly by the chromosomal collinearity with potato *StTLP*s. *MeTLP*s are at least related to abiotic stress tolerance in cassava. However, the subtle differences in function among *MeTLP*s are predictable partly because of their differential expression profiles, which are coupled with various *cis*‑acting elements existing in the promoter regions depending on genes.

## Teaser

Cassava (*Manihot esculenta* Crantz) is a high-yield starch root crop of high tolerance to poor soil conditions and abiotic stress. Tubby-like proteins (TLPs) are ubiquitous in eukaryotes and function in the abiotic stress tolerance of some plants. A cassava TLP gene, MeTLP, family containing 13 members was identified and characterized at the genomic level, showing some differences from TLP genes of other plants. The expression of MeTLPs indicates that they are related to abiotic stress tolerance. This is the first report about MeTLPs of cassava, providing clues for further study on the molecular mechanism of cassava stress resistance.

## Introduction

Tubby-like proteins (TLPs) are ubiquitous in eukaryotes (Liu 2008) which were first found to function in obese mouse (Kleyn *et al.* 1996). Thereafter, the TLPs were discovered in other species, such as human (North *et al.* 1997), chicken (Heikenwälder *et al*. 2001), rice (Kou *et al.* 2009), *Arabidopsis* (Lai *et al.* 2004), wheat (Hong *et al.* 2015) and poplar (Yang *et al.* 2008). A typical TLP protein contains a highly conserved C-terminal tubby domain that is composed of a β-barrel enclosing a central α-helix (Santagata *et al.* 2001). The tubby domain is associated with folding, solubility and subcellular localization of the TLPs (Kim *et al.* 2017). Unlike animal TLPs, the N-terminus of the TLPs from plants contains a highly conserved F-box domain in addition to the C-terminal tubby domain (Gagne *et al.* 2002; Lai *et al.* 2004). The F-box domain mediates ubiquitin proteolysis by interacting with other target proteins (Patton *et al.* 1998). The tubby and F-box domains have a co-evolutionary relationship (Kile *et al.* 2002; Yang *et al.* 2008).

More extensive functional studies have been performed on the TLPs of animals. Mutations in some TLP genes can cause obesity (Coleman *et al.* 1990; Kleyn *et al.* 1996); and vision and hearing loss, and infertility and insulin resistance (Noben-Trauth *et al.* 1996). Functional identification of the TLP genes, named *TLP*s, has been conducted only in the limited plant species. In rice, the expression of the *OsTLP* is pathogen induced (Kou *et al.* 2009). In *Arabidopsis*, *AtTLP9* and *AtTLP3* play important roles in the abscisic acid signalling pathway associated with seed germination (Bao *et al.* 2014). In chickpea, expression of *CaTLP1* is significantly upregulated under dehydration stress (Bhushan *et al.* 2007). Overexpression of *CaTLP1* in tobacco enhances the tolerance of transgenic tobacco to salt, dehydration and oxidative stresses (Wardhan *et al.* 2012). Expression of *MdTLP7* from *Malus domestica* enhances abiotic stress tolerance in *Arabidopsis* (Xu *et al.* 2019).

The number of the TLP genes in plants is much larger than in animals mainly because of segmental duplication, random translocation and insertion (Yang *et al.* 2008). It has been found that there are 11 *AtTLP*s in *Arabidopsis* (Lai *et al.* 2004), 14 *OsTLP*s in rice (Liu 2008), 11 *PtTLP*s in poplar (Yang *et al.* 2008), 4 *TaTLP*s in wheat (Hong *et al.* 2015), 15 *ZmTLP*s in maize (Chen *et al.* 2016) and 9 *MdTLP*s in apple (Xu *et al.* 2016).

Cassava (*Manihot esculenta* Crantz) is a high-yield starch root crop that is economically and socially significant (Oliveira *et al.* 2014; Perera *et al.* 2014). It has high tolerance to poor soil conditions and drought (Hu *et al.* 2016b; Wei *et al.* 2016). However, little is known about *TLP*s in cassava, named *MeTLP*s, although the genome of this crop has been sequenced and a wealth of omics data has been generated. Our hypothesis is that there is also such a *MeTLP* family in cassava, which may have some characteristics different from *TLP*s of other crops. To confirm this assumption and also to lay a foundation and provide clues for future functional identification of *MeTLP*s, in this study, we identified a cassava *MeTLP* family containing 13 genes, and further analysed chromosomal location, gene duplication, three-dimensional structure, gene structure and expression patterns of *MeTLP*s.

## Materials and Methods

### Cassava materials and public databases

The target plant species included cassava, three dicots (*Arabidopsis*, poplar and potato) and three monocots (rice, maize and sorghum). The genomic DNA sequences, coding sequences of the *TLP*s and/or amino acid sequences of these plants were obtained from the Phytozome database (https://phytozome.jgi.doe.gov/pz/portal.html).

### Genome-scale identification of MeTLPs

Unless otherwise specified, the software running parameters mentioned in the text were the default values.

The amino acid sequences of AtTLPs from the TAIR database (http://www.arabidopsis.org/) and OsTLPs from the RGAP database (http:// rice.plantbiology.msu.edu*/*) were downloaded. With amino acid sequences of the downloaded TLPs, the local HMMER model was built following the previous methods (Finn *et al.* 2011; http://www.ebi.ac.uk/Tools/hmmer/).

With the HMMER model, the data of all proteins of cassava in the Phytozome database (https://phytozome.jgi.doe.gov/pz/portal.html) were scanned to search for the candidate cassava TLPs, MeTLPs, under an *E*-value of ≤0.01. Additionally, the amino acid sequences of both AtTLPs and OsTLPs were aligned through BLASTp tool with amino acid sequences of all cassava proteins in the Phytozome database under an *E*-value of ≤0.01. Subsequently, the amino acid sequences of redundant proteins resulting from the abovementioned two approaches were removed, generating the candidate MeTLPs. All candidate MeTLPs were further verified by using two databases, CDD (Marchler-Bauer *et al.* 2017) and Pfam (Finn *et al.* 2016), under an *E*-value of ≤0.01.

The conserved domains, including the F-box domain and tubby domain, of MeTLPs were analysed by using the ClustalW (Larkin *et al.* 2007) and the BoxShade software (https://embnet.vital-it.ch/software/BOX_form.html). The phylogenetic tree was constructed using the neighbour-joining method (Tamura *et al.* 2013) by using MEGA6.0 software, with 1000 bootstrap replicates.

### Analyses of MeTLP properties and *MeTLP* structures

The molecular weight and isoelectric point of proteins were predicted by using the ExPaSy software (Artimo *et al.* 2012; http://expasy.org/). Predications for the subcellular localization of proteins were conducted through the CELLO approaches (Yu *et al.* 2006; http://cello.life.nctu.edu.tw/). The conserved amino acid sequence motifs were analysed by using the MEME software (Bailey *et al.* 2015) under the maximum 16 motifs, and then annotated by using InterProScan software (Mitchell *et al.* 2019; http://www.ebi.ac.uk/interpro/).

Intron–exon structure of the genes was analysed based on the Mesculenta_305_v6.1.gene.gff3 file of cassava from the Phytozome database by using the tool of Amazing Optional Gene Viewer function in the TBtools 0.655 (Chen *et al.* 2018; https://github.com/CJ-Chen/TBtools).

### Homologous modelling of three-dimensional structures of MeTLPs

The three-dimensional structure of MeTLPs was constructed following the homologous modelling by using the SWISS-MODEL online analysis tool (https://swissmodel.expasy.org/interactive) followed by PyMOL software-based visualization analyses.

### Analyses of gene duplication, chromosome localization and collinearity of the *TLP*s

The genome DNA file and amino acid sequences of all proteins of the target plant species in this study were downloaded from the Phytozome database and then aligned with cassava’s total proteins through the BLASTp program under an *E*-value of <10^−5^ (Kou *et al.* 2009), resulting in an m8 format output file. Both the m8 format output file and genome gff3 file_6.1 used as input files were analysed by using MCScanX software (Wang *et al.* 2012) to identify the duplicate genes. The duplicate genes were classified as five types, singleton duplication, dispersed duplication, proximal duplication, tandem duplication and segmental duplication, by using the duplicate gene classifier in MCScanX software.

Within cassava, the collinearity relationships and chromosome localization of the *MeTLP*s were drawn by using the circlize package (Gu *et al.* 2014; https://github.com/jokergoo/circlize).

Collinear maps between the *MeTLP*s and *TLP*s of other plant species were established by using the Dual Synteny Plotter tools in TBtools software (Yu *et al.* 2006; https://github.com/CJ-Chen/TBtools). Non-synonymous (*K*_a_) and synonymous (*K*_s_) rates for duplicate gene pairs were analysed by using ParaAT (Zhang *et al.* 2012) and KaKs_Calculator 2.0 software (Wang *et al.* 2010). The approximate duplicate time (*T*) of the genes was estimated following a formula (*T* = *K*_s_*/*(2*λ*), *λ* = 1.5 × 10^−8^) (Koch *et al.* 2000; Yang *et al.* 2008).

### Prediction of *cis*-acting elements in the promoter regions of *MeTLP*s

Genomic DNA fragments, 1.5 kb upstream of the gene ATG start codon, were selected as candidate promoter regions and then analysed following the approaches in the PlantCARE database (Lescot *et al.* 2002; http://bioinformatics.psb.ugent.be/webtools/plantcare/html/). The *cis*-acting elements were categorized according to a method in the literature (Hou *et al*. 2019).

### Transcriptome analysis of *MeTLP*s

As for the expression profiles of the genes in different tissues, the fragments-per-kilobase-per-million fragments mapped (FPKM) values related to cassava were download from the Gene Expression Omnibus database according to the accession number of GSE82279, which were submitted by Wilson *et al.* (2017). These data resulted from 11 tissues of 3-month-old TME 204 cassava plants that were grown in a greenhouse, including storage roots, fibrous roots, stems, petioles, leaves, lateral buds, midveins, friable embryogenic calli, somatic organized embryogenic structures, root apical meristems and shoot apical meristems.

To explore the expression profiles of the genes among different cassava varieties, the data related to two cassava cultivars (KU50 and Arg7) and one wild species W14 were downloaded from the RNA-seq read archives (SRA) (Hu *et al.* 2016b). These data resulted from 11 cassava tissues, including early storage roots (ESRs; 75 days after planting), medium tuber roots (120 days after planting) and late storage roots (150 days after planting), and stems (70 days after planting) and leaves (70 days after planting). All SRA accession numbers of the data used in this study are listed in **Supporting Information—Table S1**, and the SRA accession number-based data files were converted into FASTQ files by using the SRA Toolkit 2.9.2. The quality of the FASTQ files was verified by the FastQC 0.11.8 followed by filtering the low-quality sequences by using the Trimmomatic 0.38, resulting in the trimmed paired reads. The trimmed paired reads were aligned to the Bowtie2-indexed cassava reference genome 6.1 deposited in the Phytozome database by using the TopHat version 2.1.1 (Trapnell *et al.* 2012), resulting in the bam files of the aligned sequence reads. The gene expression level was represented by the FPKM values (Li and Dewey 2011), which were calculated based on the bam files by using the Cufflinks 2.2.1 software. The data of the FPKM values were converted into log2 values. The log2-based FPKM values were used to create the heat maps of the gene expression that were drawn through the Amazing Simple HeatMap tool in TBtools software 0.655 (Chen *et al.* 2018).

### Abiotic stress treatments of pot-grown cassava

Cassava was planted with stem cuttings strictly following the potting methods indicated in the literature (Chen *et al.* 2018). The abiotic stress treatment was conducted on 15-day old cassava plantlets after stem cutting planting. For the water deficit stress treatment, the plantlets were then transferred into a 20 % polyethylene glycol (PEG) 8000 solution and incubated for 1, 3, 6 and 9 h, respectively. For salt stress, the plantlets were then transferred into a solution containing 200 mM NaCl and incubated for 1, 3, 6 and 9 h, respectively. For low-temperature stresses, the plantlets were transferred into a growth chamber at 4 °C and stayed for 1, 3, 6 and 9 h, respectively. For phytohormone treatment, the rootlets of the plantlets were soaked for 1, 3, 6 and 9 h in 100 μM abscisic acid and in 100 μM salicylic acid solution, respectively. The controls were set up in parallel under the conditions without special treatments.

### Quantitative PCR (qPCR)

Total RNA was extracted by using a Plant RNA Kit (CWBIO, Beijing, China). One microgram of total RNA was used for the synthesis of the first-strand cDNA in a 20-µL reaction volume by using a PrimeScript™ II First-Strand cDNA Synthesis Kit (TaKaRa, Dalian, China). The reaction solution containing the first-strand cDNA was diluted 10 times with RNAase-free water and stored at −80 °C for qPCR. The qPCR was conducted with sequence-specific primers (Table 1) and the SYBR Green Mix (Vazyme, Nanjing, China) on a StepOnePlus™ Real-Time PCR System (Thermo Fisher Scientific, USA). The qPCR was performed in a 20-µL reaction volume under the thermal cycles of 95 °C for 3 s, 95 °C for 5 s and 60 °C for 30 s. The cassava gene, numbered cassava4.1_006776, was used as an internal control gene (Hu *et al.* 2016a). The relative expression level of the genes was calculated by the 2− ^ΔΔCT^ method (Livak and Schmittgen 2001), where ΔΔCt = [(Ct_target gene_ – Ct_actin gene_) under stress] – [(Ct_target gene_ – Ct_actin gene_) under control conditions]. All expression analysis of the genes had three biological replicates. Differential expression of the genes was defined at a significance level of *P* < 0.05 through Duncan’s multiple range test.

**Table 1.** qPCR primers used for analysis of expression of cassava *MeTLP*s

Gene	Forward primer	Reverse primer	Length (bp)
*MeTLP1*	GAGCCAGGCGGTTTCGTC	GGCACTGCTAAACTCGGTTG	117
*MeTLP2*	AGGAAGAACGAACACGGCAA	CAGAGACTGCATACCCTCCG	122
*MeTLP3*	TTCTGGAAGCCCCTCAGAGT	AACACGCCCTCGGAAATTCA	127
*MeTLP4*	GCACTTTAGGAGTTGTCTTCCT	TCGGTGGGCGTAGATTTCTG	118
*MeTLP5*	ACCCGAAATTATTGGCTCTCA	CCAACTTGCATCTACAACCCA	140
*MeTLP6*	TCCCAGTTGTGATGCGATTGA	CAACCGACTGACCGACTGAT	148
*MeTLP7*	CGGGCAACGAGTACCAATTT	GGATTGCATTGCTGGATGTGG	150
*MeTLP8*	CCTAGTTGAAGGTTGGGTGGG	TGTCAAGCACACAAGATTAGCA	119
*MeTLP9*	GTGGGCAAAGGTTGCTTATGG	TGCGAATGACAAAGTTCCAGTG	150
*MeTLP10*	GCTGTGGCTGACAAACATCA	AGCAAGTAGCAACAGTAACCTT	123
*MeTLP11*	ACGAGTTGAAGAGAGCGAGG	TCCGATATTGCGGGTAGACCT	121
*MeTLP12*	GGTAACACGCTGATGGGTTG	GAAGCACAGGAACCAACCTT	131
*MeTLP13*	TCCTGCCAGTGCTTCTTACA	GCTCAGGTATCACAGTCAGCA	108
Cassava4.1_006776 (internal control gene)	TGGTCAGCACATTTGTTCGT	AGCAGACCCCGTCATTGTAG	106

*MeTLP*, cassava tubby-like protein gene; qPCR, quantitative PCR.

## Results

### MeTLPs in cassava

A total of 13 cassava MeTLPs with a tubby domain were identified from the cassava genome. The MeTLPs ranged from 380 (MeTLP4) to 424 (MeTLP13 and MeTLP3) amino acids in length, 41.9 (MeTLP4) to 47.8 (MeTLP3) kDa in relative molecular mass and 8.95 (MeTLP2) to 9.62 (MeTLP13) in isoelectric point (Table 2). Detailed information on the corresponding genes (*MeTLP*s) could be fetched according to the gene ID shown in Table 2.

**Table 2.** Characteristics of *MeTLP*s and MeTLPs of cassava

*MeTLP*		MeTLP					
Name	Locus ID in Phytozome database (https://phytozome.jgi.doe.gov/pz/portal.html)	Length (amino acid residues）	Subcellular location	Amino acid residue localization of F-box domain in primary structure	Amino acid residue localization of tubby domain in the primary structure	Theoretical molecular weight (Da)	Theoretical isoelectric points
*MeTLP1*	Manes.01G214100	406	Nuclear	52–97	118–399	45 494.2	9.41
*MeTLP2*	Manes.02G095300	414	Nuclear	55–97	119–407	46 229.8	8.95
*MeTLP3*	Manes.02G179400	424	Nuclear	53–95	117–417	47 774.6	9.49
*MeTLP4*	Manes.03G100600	380	Nuclear	52–106	117–373	42 235.2	9.47
*MeTLP5*	Manes.03G190900	391	Nuclear	36–81	102–384	43 655.1	9.38
*MeTLP6*	Manes.04G100600	400	Nuclear	-	151–392	44 584.14	9.5
*MeTLP7*	Manes.05G067900	406	Nuclear	52–97	118–399	45 573	9.26
*MeTLP8*	Manes.05G157900	415	Nuclear	56–98	120–408	46 235.4	9.51
*MeTLP9*	Manes.11G069900	399	Nuclear	-	157–391	42 094.9	9.36
*MeTLP10*	Manes.15G017200	388	Nuclear	34–81	100–381	43 378.9	9.55
*MeTLP11*	Manes.15G095300	381	Nuclear	52–107	118–374	41 902	9.44
*MeTLP12*	Manes.18G023100	414	Nuclear	54–100	120–407	46 294.2	9.56
*MeTLP13*	Manes.18G091900	424	Nuclear	53–95	117–417	47 550.3	9.62

*MeTLP*, cassava tubby-like protein gene; MeTLP, cassava tubby-like protein.

### Phylogenetic evolution of TLPs

**Figure 1.**
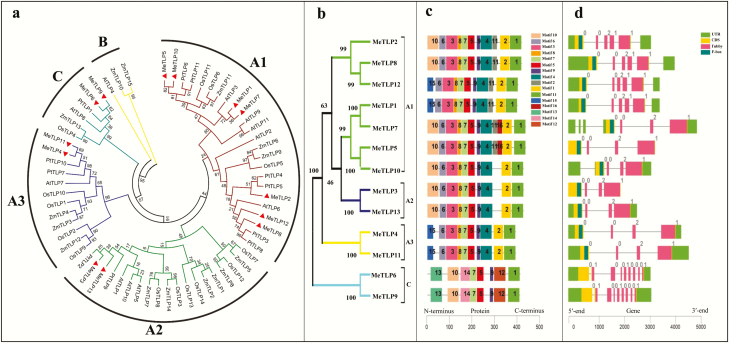
Phylogenetic trees of TLPs (a) of different plants and MeTLPs (b), conservative motifs of MeTLPs (c), and exon–intron structure of *MeTLP*s (d) in cassava.CDS, coding sequence; *MeTLP*, cassava tubby-like protein gene; MeTLP, cassava tubby-like protein; UTR, untranslated region.

A contiguous junction tree of 64 TLPs was constructed, including 13 MeTLPs, 11 AtTLPs, 15 ZmTLPs, 14 OsTLPs and 11 PtTLPs (Fig. 1a). Detailed information on these TLPs, except for cassava MeTLPs, can be found by searching with the corresponding gene ID number **[see****Supporting Information—Table S2****]**. These TLPs could be divided into three subfamilies A, B and C (Fig. 1a). The A subfamily was further divided into subgroups A1, A2 and A3, of which the A1 subgroup was largest, including 24 TLPs (5 AtTLPs, 7 MeTLPs, 3 OsTLPs, 6 PtTLPs and 3 ZmTLPs), the A2 subgroup contained 19 TLPs and the A3 subgroup was smallest, containing only 12 members. The C subfamily contained 6 TLPs. The B subfamily was smallest and contained only three TLPs (ZmTLPs 10 and 15 and AtTLP4) but did not contain any MeTLPs (Fig. 1a). Two MeTLPs 6 and 9 that were categorized into the subfamilies C were found to be on an evolutionary branch different from that of the other 11 MeTLPs (Fig. 1a).

### Putative conserved sequence motifs of MeTLPs and genomic structure of *MeTLP*s

A total of 16 conserved amino acid sequence motifs were found in MeTLPs, including 1 F-box domain (motif 3) in the N-terminus, six tubby domains (motifs 1, 2, 4, 5, 7 and 12) in the C-terminus, and nine unknown motifs (motifs 6, 8–10, 11 and 13–16) (Fig. 1b; **Supporting Information—Table S3**). In general, the same subfamily of MeTLPs (Fig. 1b) shared similar putative conserved sequence motifs (Fig. 1c). Eleven MeTLPs 1–5, 7, 8 and 10–13 had both F-box and tubby domains, and two MeTLPs 6 and 9 had no F-box domains. All the MeTLPs had three tubby domains of motifs 1, 5 and 7. The tubby domains of motif 12 were present in only MeTLPs 6 and 9 (Fig. 1c; **Supporting Information—Table S3**).

Eleven *MeTLP*s in the A subfamily (Fig. 1a and b) contained three to six introns (Fig. 1d). Two *MeTLP*s 6 and 9 in the C subfamily (Fig. 1a and b) had eight introns (Fig. 1d).

**Figure 2.**
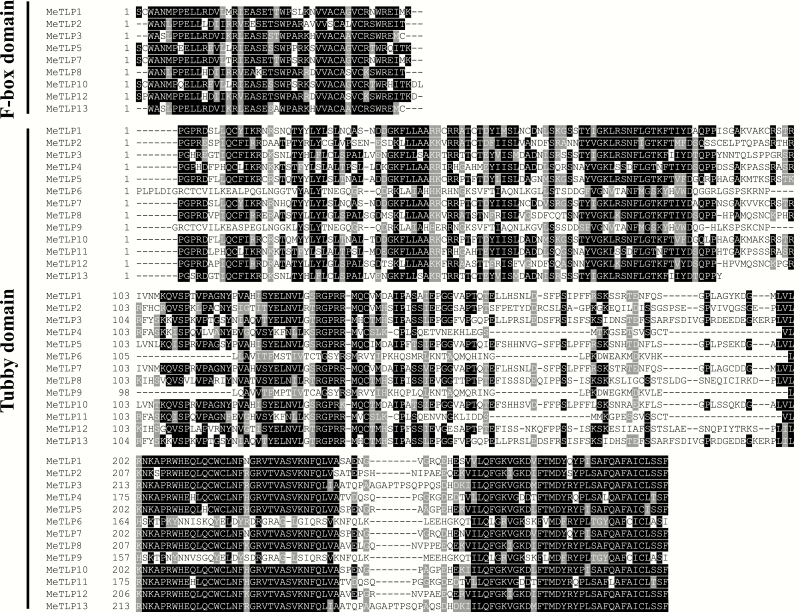
Conserved amino acid residues in the tubby domain and F-box domain of MeTLPs. MeTLP, cassava tubby-like protein.

The conserved amino acid residues in F-Box and tubby domains of MeTLPs were shown in Fig. 2.

### Chromosomal distribution and collinearity of *MeTLP*s

**Figure 3.**
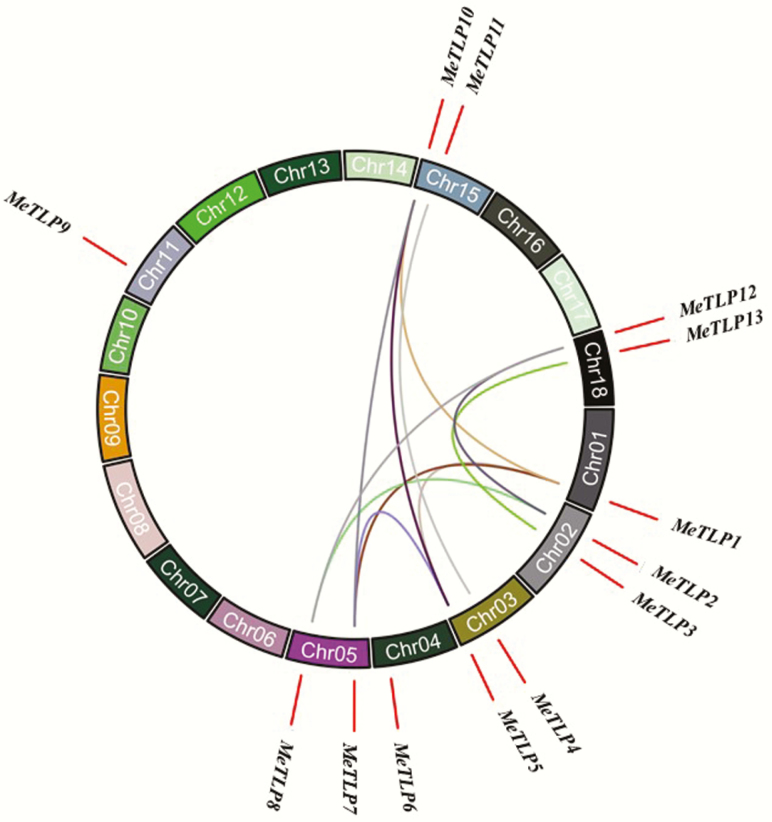
Chromosomal distribution and duplication of *MeTLP*s in the cassava genome. The coloured curves indicate segmental duplication genes. *MeTLP*, cassava tubby-like protein gene.

A total of 13 *MeTLP*s were mapped to seven of the 18 cassava chromosomes (Fig. 3). Eleven pairs of *MeTLP* duplications, which belonged to whole-genome duplications/segmental duplications, were found **[see****Supporting Information—Table S4****]**. The *K*_a_/*K*_s_ values of 11 pairs of *MeTLP* duplications were all <1, therefore, their duplication likely took place between 10.11 million years and 126.69 million years ago **[see****Supporting Information—Table S4****]**.

**Figure 4.**
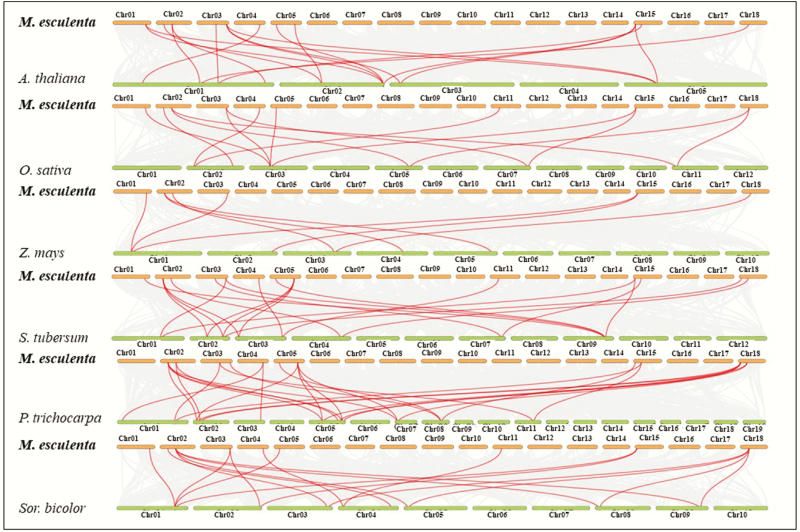
Collinearity between cassava *MeTLP*s and *TLP*s from other plants. The grey lines in the background indicate the collinear blocks within the genomes of cassava and other plants, whereas the red lines highlight the syntenic *TLP* pairs. The Arabic numerals after Chr, such as 01, indicated chromosome number. *A*., *Arabidopsis*; Chr., chromosome; *M*., *Manihot*; *MeTLP*, cassava tubby-like protein gene; *O*., *Oryza*; *P*., *Populus*; *S*., *Solanum*; *Sor*., *Sorghum*; *Z*., *Zea*.

There were 11, 11 and 13 *MeTLP*s that showed collinearity with the *TLP*s of three dicots, *Arabidopsis*, poplar and potato (Fig. 4; **Supporting Information—Table S5**), respectively. There were 9, 6 and 9 *MeTLP*s that had collinearity with the *TLP*s of three monocots, rice, maize and sorghum (Fig. 4; **Supporting Information—Table S5**), respectively. Interestingly, five *MeTLP*s *2*, *3*, *5*, *10* and *13* had collinearity with the *TLP*s from both dicots and monocots (Fig. 4; **Supporting Information—Table S5**). Three *MeTLP*s *4*, *8* and *11* showed collinearity with the *TLP*s of only three dicots, *Arabidopsis*, poplar and potatoes (Fig. 4; **Supporting Information—Table S5**), respectively.

### Three-dimensional structure of MeTLPs

**Figure 5.**
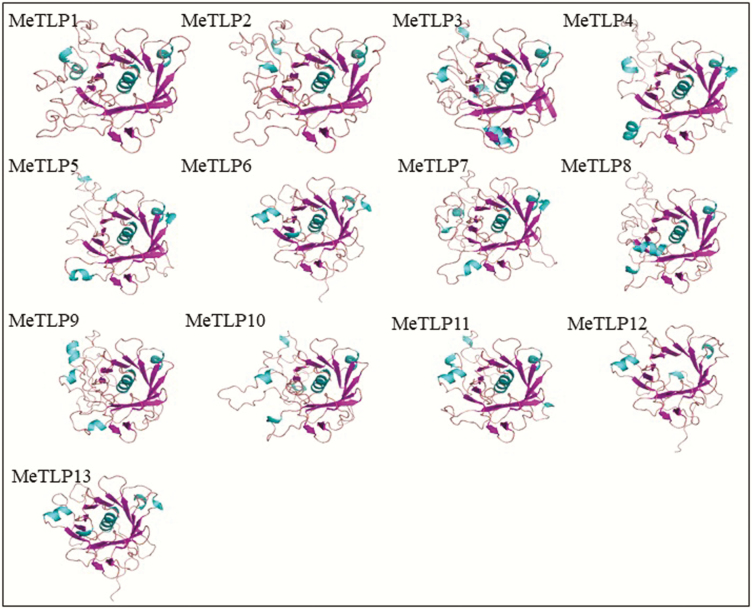
Homology modelling of the three-dimensional structure of MeTLPs. The α helixes are shown in green, and β-folds are shown in purple. MeTLP, cassava tubby-like protein.

As shown in Fig. 5, nine MeTLPs 1, 2, 4, 5–8, 11 and 13 presented a typical tubby architecture formed by a closed β-barrel that was composed of one central α-helix and 12 anti-parallel strands. Three MeTLPs 3, 9 and 10 contained an incomplete β-barrel. MeTLP3 had one central α-helix and 11 antiparallel strands. Two MeTLPs 9 and 10 showed one central α-helix and 10 anti-parallel strands. MeTLP12 just had 12 antiparallel strands but no central α-helixes.

### *Cis*-acting elements in potential promoter regions of *MeTLP*s

A total of 68 *cis*-acting elements were found in potential promoter regions of 13 *MeTLP*s, including 28 functionally unknown elements. These *cis*-acting elements could be divided into the following seven types (Table 3; **Supporting Information—Table S6**): development, environmental stress, hormone-responsive, light-responsive, promoter-related, site-binding and other aspects. Of the *cis*-acting elements, the number of light-responsive elements was up to 16, accounting for 23.9 % of the total *cis*-acting elements.

**Table 3.** The number and types of *cis*-acting elements in the promoter regions of *MeTLP*s

*MeTLP*	Development	Environmental stress	Hormone responsive	Light responsive	Promoter related	Site binding	Others
*MeTLP1*	0	2	5	9	2	0	10
*MeTLP2*	0	3	7	4	2	0	12
*MeTLP3*	1	3	2	6	2	0	9
*MeTLP4*	0	4	2	3	2	0	9
*MeTLP5*	0	2	3	7	2	0	10
*MeTLP6*	1	1	5	4	2	0	8
*MeTLP7*	0	2	3	6	2	0	8
*MeTLP8*	0	3	3	4	2	0	15
*MeTLP9*	1	3	2	6	2	0	8
*MeTLP10*	1	2	6	7	2	1	11
*MeTLP11*	1	5	5	4	2	2	11
*MeTLP12*	0	0	0	1	2	0	0
*MeTLP13*	1	3	2	5	2	1	8

*MeTLP*, cassava tubby-like protein gene.

For the development-related elements (Table 3; **Supporting Information—Table S6**), they were found in the promoters of six *MeTLP*s not including *MeTLP*s *1*, 2, *4*, *5*, *7*, *8* and *12*, which could be subdivided into the following groups: CAT-box and CCGTCC-box related to the development of meristems; GCN4 motif relevant to the development of endosperm; and RY-element associated with seed-specific regulation.

Of six environmental stress-related elements (Table 3; **Supporting Information—Table S6**), both anaerobic induction (ARE) and GC motifs were essential for anaerobic induction, of which AREs existed in the promoters of 11 *MeTLP*s. Low-temperature responsiveness and MYB-binding sites (MBSs) were related to the plant response to low temperature and drought, respectively. LTRs responsive to low temperature were found in the promoters of *MeTLP*s *1*, *2*, *4*, *9*, *11* and *13*. MBSs were found in *MeTLP*s *3*, *4*, *11* and *13*. No abiotic stress-related elements were found in the promoters of *MeTLP12*.

Of 10 hormone-responsive elements (Table 3; **Supporting Information—Table S6**), both AuxRR-core and TGA elements were responsive to auxin, and abscisic acid-responsive element (ABRE) responded to abscisic acid. Both CGTCA motifs and TGACG motifs were responsible to methyl jasmonate. Both gibberellin responsive element motifs and P-boxes responded to gibberellin. TCA motifs and ethylene-responsive elements (EREs) were associated with salicylic acid and ethylene, respectively. One ERE was present in the promoters of 9 *MeTLP*s *2*,*4*–*7*, *9*–*11* and *13*. One ABRE was present in the promoters of eight *MeTLP*s *1*, *3*, *4*–*6*, *8*, *10* and *11*. No hormone-responsive elements were found in the promoters of *MeTLP12*.

### Expression patterns of *MeTLP*s based on public transcriptome databases

**Figure 6.**
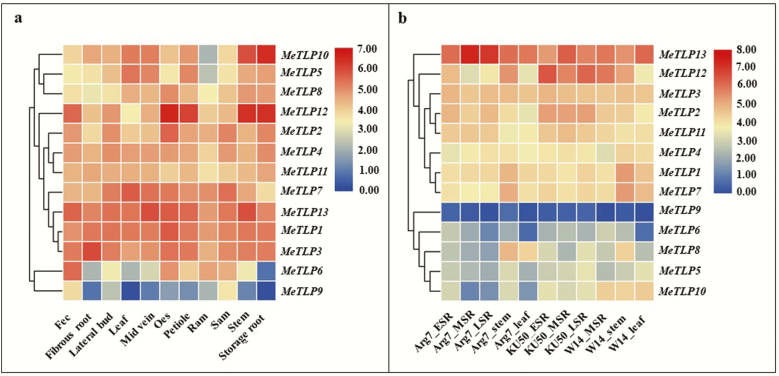
Heat maps of expression of *MeTLP*s in 11 tissues of 3-month-old TME 204 cassava (a) and in the tissues among two cassava cultivars of Arg7 and KU50, and cassava wild species W14 (b). ESR, early storage root; Fec, friable embryogenic callus; LSR, last storage root; *MeTLP*, cassava tubby-like protein gene; MSR, middle storage root; Oes, somatic organized embryogenic structure; Ram, root apical meristem; Sam, shoot apical meristem.

There were 10 *MeTLP*s, except *MeTLP*s *6*, *9* and *10*, which had a high expression level in 11 tissues of 3-month-old TME 204 cassava (Wilson *et al.* 2017). *MeTLP6* showed a high expression level in eight cassava tissues except for fibrous and storage roots, and leaves. *MeTLP9* had a high expression level in only friable embryogenic calli and shoot apical meristems. *MeTLP10* showed a high expression level in 10 tissues except for root apical meristems (Fig. 6a; **Supporting Information—Table S7**).

Using data in the high-throughput SRA (Hu *et al.* 2016b), expression of *MeTLP*s in wild species W14 and two cultivars (KU50 and Arg7) was analysed (Fig. 6b). Five *MeTLP*s *1*, *3*, *7*, *11* and *13* had a high expression level in all tissues of these cassava materials. Three *MeTLP*s *5*, *6* and *9* showed a low expression level in all cassava tissues. *MeTLP2* had a low expression level only in Arg7 leaves, *MeTLP4* had a low expression level only in ESRs of Arg7 and middle storage roots (MSRs) of W14. *MeTLP8* had a high expression level only in stems and leaves of Arg7 and stems of W14. *MeTLP10* had a high expression level only in MSRs, stems and leaves of W14. *MeTLP12* had a low expression level only in ESRs and leaves of Arg7 (Fig. 6b; **Supporting Information—Table S8**).

## Expression of *MeTLP*s in rootlets pot-grown cassava Arg7 in response to abiotic stresses, abscisic acid and salicylic acid

**Figure 7.**
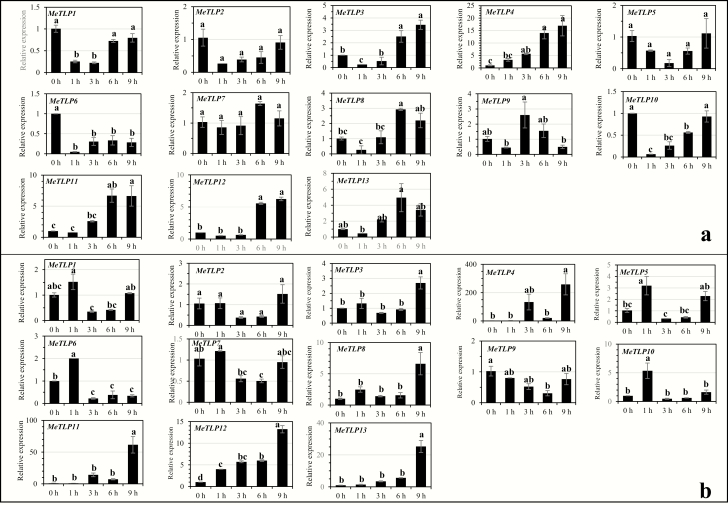
Expression of *MeTLP*s in roots of plantlets of pot-grown cassava Arg7 under 200 mM NaCl (a) and 20 % PEG (b) stress. The expression of *MeTLP*s in roots of 15-day-old cassava plantlets of cassava Arg7 after stem cutting planting was analysed by qPCR. Three biological repeats were conducted. Different letters on the columns indicate a statistically significant difference at a significance level of *P* < 0.05 by Duncan’s multiple range test. *MeTLP*, cassava tubby-like protein gene; qPCR, quantitative PCR. PEG, polyethylene glycol.

**Figure 8.**
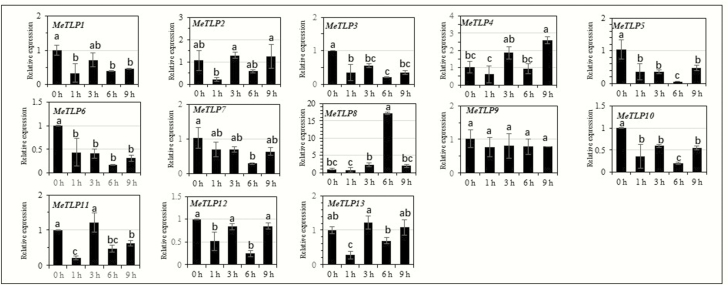
Expression of *MeTLP*s in roots of plantlets of pot-grown cassava Arg7 under a low temperature at 4 °C. The expression of *MeTLP*s in roots of 15-day-old cassava plantlets of cassava Arg7 after stem cutting planting was analysed by qPCR. Three biological repeats were conducted. Different letters on the columns indicate a statistically significant difference at a significance level of *P* < 0.05 by Duncan’s multiple range test. *MeTLP*, cassava tubby-like protein gene; qPCR, quantitative PCR.

**Figure 9.**
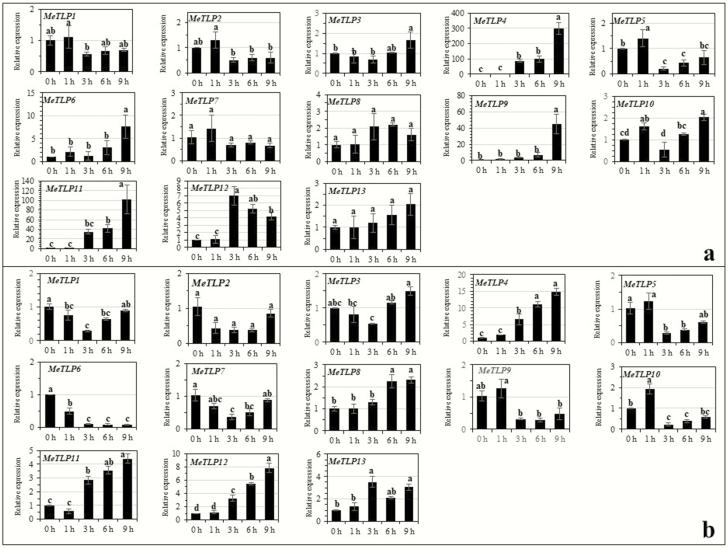
Expression of *MeTLP*s in roots of plantlets of pot-grown cassava Arg7 in response to 100 μM abscisic acid (a) and 100 μM salicylic acid (b). The expression of *MeTLP*s in roots of 15-day-old cassava plantlets of cassava Arg7 after stem cutting planting was analysed by qPCR. Three biological repeats were conducted. Different letters on the columns indicate a statistically significant difference at a significance level of *P* < 0.05 by Duncan’s multiple range test. *MeTLP*, cassava tubby-like protein gene; qPCR, quantitative PCR.

Under NaCl treatment (Fig. 7a), expression of *MeTLP*s could be grouped into the following patterns: significantly upregulated at or after 3 h of the stress, as was the case for *MeTLP*s *3*, *4*, *8, 11* and *12*; significantly downregulated throughout the stress, as was the case for only *MeTLP6*; and unchanged throughout the stress, as was the case for *MeTLP*s *2*, *5* and *7*. Under PEG treatment (Fig. 7b), expression of *MeTLP*s could be grouped into the following patterns: significantly upregulated at only 1 h of the stress, as was the case for *MeTLP*s *5*, *6* and *10*; significantly upregulated throughout the stress, as was the case for only *MeTLP12*; significantly upregulated at only 9 h of the stress, as was the case for *MeTLP*s *3*, *8*, *11* and *13*; and unchanged throughout the stress, as was the case for only *MeTLP2.* Under low-temperature treatment (Fig. 8), expression of *MeTLP*s could be grouped into the following patterns: significantly downregulated throughout the stress, as was the case for *MeTLP*s *3*, *5*, *6* and *10*; and unchanged throughout the stress, as was the case for only *MeTLP9.* Under abscisic acid treatment (Fig. 9a), expression of *MeTLP*s could be grouped into the following patterns: significantly upregulated at or after 3 h of treatment, as was the case for *MeTLP*s *4*, *11* and *12*; significantly upregulated at 9 h of treatment, as was the case for *MeTLP*s *3*, *6* and *9*; and unchanged throughout abscisic acid treatment, as was the case for *MeTLP*s *7*, *8* and *13*. Under salicylic acid treatment (Fig. 9b), expression of *MeTLP*s could be grouped into the following patterns: significantly upregulated at or after 3 h of treatment, as was the case for *MeTLP*s *4*, *8*, *11* and *12*; significantly downregulated throughout the treatment, as was the case for only *MeTLP6*; and unchanged throughout salicylic acid treatment, as was the case for only *MeTLP2*.

## Discussion

It has been known that cassava is highly tolerant to drought and soil infertility (Hu *et al.* 2016b; Wei *et al.* 2016). Reportedly, *TLP*s play an important role in plant growth, development and abiotic stress responses (Bao *et al.* 2014; Du *et al.* 2014; Xu *et al.* 2016). In this study, a total of 13 *MeTLP*s were identified in cassava, different in number from that of *TLP*s of other plants (Lai *et al.* 2004; Yang *et al.* 2008; Hong *et al.* 2015; Chen *et al.* 2016; Xu *et al.* 2016).

The TLPs from maize, poplar, *Arabidopsis* and rice were evolutionally classified into three subfamilies of A, B and C (Fig. 1), being in accordance with previous reports in *Arabidopsis* (Yang *et al.* 2008), apple (Xu *et al.* 2016) and maize (Chen *et al.* 2016). However, no MeTLPs in the B subfamily suggests that the functional evolution of *MeTLP*s is not exactly the same as that of *Arabidopsis*, apple and maize.

It should be pointed out that the β-barrel of MeTLPs 3, 9 and 10 was incomplete (Fig. 5), showing findings analogous to those in apples (Xu *et al.* 2016). According to classification of the TLP family (Wang *et al.* 2018), 14 MeTLPs 1–5, 7, 8 and 10–16 should belong to class III because they had both the tubby domain and F-box domain (Fig. 1b and c),and MeTLPs 6 and 9 from the C subfamily may be assigned to class II because they do not have the F-box domain in the N-terminus, different from the finding in Arabidopsis that only AtTLP8 out of the AtTLPs does not contain the conserved F-box domain (Lai *et al.* 2004), also not fully agreeing with previous reports that the tubby and F-box domains have a co-evolutionary relationship (Kile *et al.* 2002; Yang *et al.* 2008). However, the results could suggest that the two corresponding genes, *MeTLP*s *6* and *9*, likely originate from one gene in an ancestral species and may be common ancestors for other *MeTLP*s.

Similar to the findings in other species (Yang *et al.* 2008; Du *et al.* 2014), the *MeTLP*s belonging to the same subfamily had similar exon–intron structures (Fig. 1b and d) and the corresponding MeTLPs possessed similar conserved amino acid sequence motifs **[see****Supporting Information—Table S3****]**, suggesting that there were similar but redundant functions among these genes. Of 16 conserved sequence motifs, three tubby domains of motifs 1, 5 and 7 were found in all MeTLPs (Fig. 1b and c), indicating the signatures of the tubby domains (Lai *et al.* 2004; Kou *et al.* 2009; Xu *et al.* 2016).

 Gene duplication is also known as the source of differentiation in novel genes during evolution (Moore and Purugganan 2003; Kong *et al.* 2007). A total of 11 pairs of segmental duplications of *MeTLP*s **[see****Supporting Information—Table S4****]** were different from five pairs of segmental duplications of *OsTLP*s in rice and six pairs of segmental duplications of *PtTLP*s in poplar (Yang *et al.* 2008), respectively, indicating that segmental duplication, rather than other duplications, is more important for *MeTLP* duplications in cassava than for *TLP* duplication in rice and poplar.

The collinearity of *MeTLP*s with the *TLP*s of six other representative plant species (Fig. 4; **Supporting Information—Table S5**) provides a reference for cloning and functional studies of cassava *MeTLP*s. More *MeTLP*s showed collinearity with potato *StTLP*s (Fig. 4; **Supporting Information—Table S5**), suggesting that these two types of *TLP*s have the conserved gene order within the corresponding chromosomal segment in these two crops (Keller and Feuillet 2000). *MeTLP*s *2*, *3*, *5*, *10* and *13* showed collinearity with *TLP*s of analysed plant species (Fig. 4; **Supporting Information—Table S5**), indicating that these five orthologous pairs had already existed before the divergence of dicots and monocots, which is a phenomenon similar to the duplication of the WRKY gene family in pineapples (Xie *et al.* 2018). *MeTLP*s *4*, *8* and *11* showed collinearity with the *TLP*s of three dicots, *Arabidopsis,* poplar, and potato, indicating that they occurred after the divergence of dicots.

The TLPs from different classes are very different in function (Lai *et al.* 2012). The F-box family proteins are characterized by a signature of the F-box domain (Jia *et al.* 2013). So, MeTLPs 1–5, 7, 8 and 10–16 can also be simultaneously categorized as the F-box family.

The F-box proteins are involved in ubiquitin-dependent proteolysis in cell cycle regulation and signal transduction, which function in many aspects such as cell elongation and division, injury response, floral differentiation, circadian clock and response to the plant growth regulators auxin and jasmonic acid in plants (Craig and Tyers 1999; del Pozo and Estelle 2000; Ho *et al.* 2008; Baute *et al.* 2017). Relatively to the F-box proteins, most plant TLPs remain a mystery in function although they have been found across plant species (Lai *et al.* 2012; Wang *et al.* 2018). The limited reports indicate that plant TLPs play roles in responses to multifarious stresses including biotic and abiotic stress (Bhushan *et al.* 2007; Kou *et al.* 2009; Wardhan *et al.* 2012; Bao *et al.* 2014; Wang *et al.* 2018; Xu *et al.* 2019). Two *MeTLP*s *6* and *9* would most likely not be eligible for ubiquitin-related protein degradation in the early stages of evolution because their corresponding MeTLPs lack the F-box domain (Fig. 1d; Table 2).

 The increases in abiotic stressors are important constraints for food production and farming worldwide, including drought/water deficit, salt, and cold/low temperature (Roychoudhury *et al.* 2013; Razzaq *et al.* 2019). The tolerance to abiotic stressors in plants depends on some abiotic stress-responsive genes and phytohormone signalling pathways such as abscisic acid (Roychoudhury *et al.* 2013) and salicylic acid (Eremina *et al.* 2016). The changes in expression profiles with the tissues (Fig. 6) as well as expression responses to salt and PEG (Fig. 7), low temperature (Fig. 8), and abscisic acid and salicylic acid (Fig. 9) suggest that, like the *TLP*s in other plants, *MeTLP*s may make contributions to abiotic stress tolerance in cassava. Although further functional validation is required, subtle differences in function among *MeTLP*s are predictable partly because of their differential expression profiles depending on cassava cultivars and tissues (Fig. 6) and/or under different treatments (Fig. 7). The part of the reason for this is likely related to various *cis*‑acting elements existing in the promoter regions depending on genes (Table 3; **Supporting Information—Table S6**).

## CONCLUSION

There is a *MeTLP* family containing 13 members, which can be divided into A and C subfamilies. There are 11 pairs of *MeTLP*s that show the duplication which likely took place between 10.11 and 126.69 million years ago. Two *MeTLP*s *6* and *9* likely originate from one gene in an ancestral species, may be common ancestors for other *MeTLP*s, and their corresponding proteins (MeTLPs 6 and 9) would most likely not be eligible for ubiquitin-related protein degradation in the early stages of evolution because they just have the tubby domain in the C-terminus but no the F-box domain in the N-terminus. *MeTLP*s feature differences in the number from *TLP*s in wheat, apple, *Arabidopsis*, poplar and maize, and are highlighted by segmental duplication but more importantly by the chromosomal collinearity with potato *StTLP*s. Like *TLP*s in other plants, *MeTLP*s are at least related to abiotic stress tolerance in cassava. However, the subtle differences in function among *MeTLP*s are predictable partly because of their differential expression profiles depending on cassava cultivars and tissues and/or under different treatments, which are coupled with various *cis*‑acting elements existing in the promoter regions depending on genes.

## SUPPORTING INFORMATION

The following additional information is available in the online version of this article —

**Table S1.** The accession number of the public high-throughput RNA-seq read archives databases submitted by Hu *et al.* (2016b).

**Table S2.** The ID number for *TLP*s in plant species analysed in this study.

**Table S3.** The amino acid sequences and annotation of conserved motifs of MeTLPs.

**Table S4.** The segmental duplication events of cassava *MeTLP* family.

**Table S5.** The pair relationships of orthologous between cassava *MeTLP*s and *TLP*s of other plants.

**Table S6.** The potential *cis*-acting elements in the promoter region of cassava *MeTLP*s.

**Table S7.** The expression profiles of cassava *MeTLP*s in 11 tissues of 3-month-old TME 204 cassava plants based on the GEO database submitted by Wilson *et al.* (2017).

**Table S8.** The expression of cassava *MeTLP*s in different tissues of cultivars Ku50 and Arg7, and wild species W14 based on the high-throughput SRA databases submitted by Hu *et al.* (2016b).

plz075_suppl_Supplementary_Information_of_Tables_S2-S7

plz075_suppl_Supplementary_Table_S1

plz075_suppl_Supplementary_Table_S8

## Sources of Funding

This work was supported by the Innovation Project of Guangxi Graduate Education in 2018 (YCBZ2018020) from the Department of Education of Guangxi Zhuang Autonomous Region, Guangxi, China; and the funding (SKLCUSA-a201804) from the State Key Laboratory for Conservation and Utilization of Subtropical Agro-bioresources, Guangxi Univiersity, Guangxi, China.

## Contributions by the Authors

M.-Y.D. conducted data analysis and experiments and wrote the first draft. X.-W.F. assisted in the design and management of experiments. X.-Y.P. helped to conduct the gene expression experiments. Y.-Z.L. conceived the project, designed the experiments and wrote the manuscript as a supervisor.

## Conflicts of Interest

None declared.
