# Supplementary material for: Decrypting tubby-like protein gene family of multiple functions in starch root crop cassava
Source: AoB Plants. 2019 Nov 25;11(6):plz075. doi: 10.1093/aobpla/plz075 (PMC6920310; doi:10.1093/aobpla/plz075)
Supplement: plz075_suppl_Supplementary_Information_of_Tables_S2-S7 [file plz075_suppl_supplementary_information_of_tables_s2-s7.pdf]

Table S2 The ID number for *TLP*s in plant species analysed in this study

AtTLPs* of *Arabidopsis		***OsTLPs* of rice**		***ZmTLPs* of maize**		***PtTLPs* of poplar**	
**Name**	**ID**	**Name**	**ID**	**Name**	**ID**	**Name**	**ID**
AtTLP1	At1g76900.1	*OsTLP1*	LOC_Os01g48370.1	*ZmTLP1*	GRMZM2G001272_T01	*PtTLP1*	Potri.001G041400.1
AtTLP2	At2g18280.1	*OsTLP2*	LOC_Os01g55430.1	*ZmTLP2*	GRMZM2G046816_T01	*PtTLP2*	Potri.002G068200.4
AtTLP3	At2g47900.1	*OsTLP3*	LOC_Os01g64700.1	*ZmTLP3*	GRMZM2G062154_T01	*PtTLP3*	Potri.005G228500.1
AtTLP4	At1g61940.1	*OsTLP4*	LOC_Os02g08310.1	*ZmTLP4*	GRMZM2G068586_T01	*PtTLP4*	Potri.007G023400.1
AtTLP5	At1g43640.1	*OsTLP5*	LOC_Os02g47640.1	*ZmTLP5*	GRMZM2G108228_T01	*PtTLP5*	Potri.005G121600.1
AtTLP6	At1g47270.1	*OsTLP6*	LOC_Os03g22800.1	*ZmTLP6*	GRMZM2G115701_T01	*PtTLP6*	Potri.010G031000.1
AtTLP7	At1g53320.1	*OsTLP7*	LOC_Os04g59130.1	*ZmTLP7*	GRMZM2G129288_T01	*PtTLP7*	Potri.011G109300.1
AtTLP8	At1g16070.1	*OsTLP8*	LOC_Os05g36190.1	*ZmTLP8*	GRMZM2G163726_T01	*PtTLP8*	Potri.002G034600.1
AtTLP9	At3g06380.1	*OsTLP9*	LOC_Os05g43850.1	*ZmTLP9*	GRMZM2G176340_T01	*PtTLP9*	Potri.005G192300.2
AtTLP10	At1g25280.1	*OsTLP10*	LOC_Os05g48670.1	*ZmTLP10*	GRMZM2G349376_T01	*PtTLP10*	Potri.001G390200.1
AtTLP11	At5g18680.1	*OsTLP11*	LOC_Os07g47110.1	*ZmTLP11*	GRMZM2G378907_T01	*PtTLP11*	Potri.008G195200.1
		OsTLP12	LOC_Os08g01290.1	*ZmTLP12*	GRMZM2G435445_T01		
		OsTLP13	LOC_Os11g06420.1	*ZmTLP13*	GRMZM2G472945_T01		
		OsTLP14	LOC_Os12g06630.1	*ZmTLP14*	GRMZM5G866954_T01		
				ZmTLP15	GRMZM5G871407_T01		

TLP, tubby-like protein gene

Table S3 The amino acid sequences, and annotation of conserved motifs of MeTLPs

**Motif**	**E-value**	**Start site**	**Length (amino acid residues)**	**Consensus sequence**	**Annotation**
Motif 1	3.1E-392	13	50	[GN][VP][GPA]XE[ED][HQ][ED]K[VI]ILQFGK[VI]GKDIFTMDYRYP[LI]SAFQAFAICLSSFDTK[LIP]ACE	Tubby, C-terminal
Motif 2	4.0E-335	11	41	PLVL[RK]NKAPRWHEQLQCWCLNF[HR]GRVTVASVKNFQLVA[AS][VPT]E	Tubby, C-terminal
Motif 3	7.0E-317	11	50	VVACAGVC[RK]SWREITKEIVK[TS]PE[FQ]SG[KR][LI]TFP[IV]SLKQPGPRD[FS][PL][IL]QCFI[KR]R	F-box domain
Motif 4	1.80E-262	11	50	VPAG[NS]Y[NP][VI][AG][HQT][VI][ST]YELNVL[GR][ST]RGPRRMQC[TV]M[HD][SA]IPASSIEPGGVAP[TG][QP]TE[FL]	Tubby, C-terminal
Motif 5	3.00E-174	13	29	[DG][DN][LF]S[QRK][GS]S[SN][TA]Y[VI]GKLRSNFLGTKFT[IV][YF]DSQ	Tubby, C-terminal
Motif 6	4.50E-159	11	29	SCWA[NS][ML]PPELLR[DE][VI][IL]KR[VI]E[AE]SE[TS][ST]WP[AS]RK	-
Motif 7	1.90E-121	13	29	[ALS][LS][GN][DE]D[GD]K[FL]LLAA[KRH][RK]CR[RH][AP][TA]CT[DE][YF]IISL[DNV]A	Tubby, C-terminal
Motif 8	3.20E-45	11	15	[DN][RK][SA][TN][QS]TYYLYL[SG]L[ST]P	-
Motif 9	2.50E-27	13	15	[KS][SC]RS[ST]RR[FV][NH]SK[QK][VI]SP	-
Motif 10	9.60E-57	9	49	[ML][SK][FLP][KRT][SG][IFL][VLQ][RFQ][DEC][ML][RQ][EDGM][GIN][FER][GP][SDE][ILMR][SA][RL][KIR][SG][FI][DGL][KLVW][DKR][FHL][GT][KEG][GHS][MEH][RK][SGR][RAK][SAG][HRS][SALR][HNSV][IV][AQW][DPE][ELY][SG][DF][SQ][PE][VAL][ADPV][AIP][AEFQ]	-
Motif 11	6.20E-23	6	21	[HR][SH][NL][ELV]DSF[PR]S[IL][PS]F[FS][RK]S[KI][SD]N[RS]TE	-
Motif 12	8.90E-20	2	50	NTAQMQ[HR]INGLPKDWE[AG]KMDKV[HL][EK]LHSKTP[KN]YNN[IV]S[GK]QYELDY[RS]DRGRAG	Tubby, C-terminal
Motif 13	4.10E-15	2	50	YN[CS][LV]Y[LV][DN]PLTD[LP]KHHH[GS]CSEGDL[AT][AT]VFTDNKENNPIFG[NS][DG]KENA[IV]PKSSN	-
Motif 14	7.00E-09	2	41	NR[AS]LLCR[AP]LPLDIGRCTCVILKEA[LS]P[EQ]GLNGG[KT][LV]Y[AS]LYTNE	-
Motif 15	2.10E-04	4	26	MS[AL][IR][KR]S[IM][IL][ICS]R[RS][IR]S[HNR][RS][VF][VKR][SQR][DV][GN][NPS][AH][KAS][HAE][PDG][EDG]	-
Motif 16	1.80E-02	2	21	FSSARFSDIVGPRD[EG]EDEGKE	-

MeTLP, cassava TLP protein； TLP, tubby-like protein

Table S4 The segmental duplication events of cassava *MeTLP* family

Duplicated pairs		Ka	Ks	Ka/Ks	Estimated duplicate time (million year)	Duplicate type
MeTLP1	*MeTLP5*	0.16	1.82	0.09	60.71	WGDs/segmental
MeTLP1	*MeTLP7*	0.04	0.34	0.11	11.23	WGDs/segmental
MeTLP1	*MeTLP10*	0.16	1.76	0.09	58.62	WGDs/segmental
MeTLP2	*MeTLP8*	0.25	3.08	0.08	102.81	WGDs/segmental
MeTLP3	*MeTLP13*	0.02	0.41	0.05	13.76	WGDs/segmental
MeTLP2	*MeTLP12*	0.23	3.80	0.06	126.69	WGDs/segmental
MeTLP5	*MeTLP7*	0.16	1.28	0.12	42.79	WGDs/segmental
MeTLP5	*MeTLP10*	0.06	0.30	0.19	10.11	WGDs/segmental
MeTLP4	*MeTLP11*	0.07	0.33	0.21	11.01	WGDs/segmental
MeTLP7	*MeTLP10*	0.16	1.40	0.11	46.75	WGDs/segmental
MeTLP8	*MeTLP12*	0.08	0.40	0.21	13.24	WGDs/segmental

Ka, non-synonymous rate; Ks, synonymous rate; *MeTLP*, cassava tubby-like gene; TLP, tubby-like protein; WGDs, whole-genome duplications

Table S5 The pair relationships of orthologous between cassava *MeTLP*s and *TLP*s of other plants

***TLP* pair between cassava and dicots**

TLP* pair between cassava and *Arabidopsis		***TLP* pair between cassava and poplar**		***TLP* pair between cassava and potato**	
MeTLP	***AtTLP* ID**	***MeTLP***	***PtTLP* ID**	***MeTLP***	***StTLP* ID**
MeTLP6	AT1G16070.2	*MeTLP6*	Potri.001G041400.1	*MeTLP1*	PGSC0003DMT400029513
MeTLP13	AT1G43640.1	*MeTLP4*	Potri.001G390200.1	*MeTLP2*	PGSC0003DMT400009281
MeTLP4	AT1G53320.1	*MeTLP11*	Potri.001G390200.1	*MeTLP3*	PGSC0003DMT400061933
MeTLP11	AT1G53320.1	*MeTLP2*	Potri.002G034600.1	*MeTLP4*	PGSC0003DMT400018209
MeTLP3	AT1G76900.1	*MeTLP8*	Potri.002G034600.1	*MeTLP5*	PGSC0003DMT400027634
MeTLP2	AT2G18280.1	*MeTLP12*	Potri.002G034600.1	*MeTLP6*	PGSC0003DMT400036862
MeTLP8	AT2G18280.1	*MeTLP3*	Potri.002G068200.1	*MeTLP7*	PGSC0003DMT400029513
MeTLP1	AT2G47900.3	*MeTLP13*	Potri.002G068200.1	*MeTLP8*	PGSC0003DMT400009281
MeTLP5	AT2G47900.3	*MeTLP5*	Potri.008G195200.1	*MeTLP9*	PGSC0003DMT400036862
MeTLP7	AT2G47900.3	*MeTLP7*	Potri.008G195200.1	*MeTLP10*	PGSC0003DMT400027634
MeTLP10	AT2G47900.3	*MeTLP10*	Potri.008G195200.1	*MeTLP11*	PGSC0003DMT400018209
				MeTLP12	PGSC0003DMT400009281
				MeTLP13	PGSC0003DMT400061933

***TLP* pair between cassava and monocots**

***TLP* pair between cassava and rice**		***TLP* pair between cassava and maize**		***TLP* pair between cassava and sorghum**	
MeTLP	***OsTLP* ID**	***MeTLP***	***ZmTLP* ID**	***MeTLP***	***SbTLP* ID**
MeTLP3	LOC_Os01g64700.2	*MeTLP2*	GRMZM2G176340_T01	*MeTLP1*	Sobic.001G372000.1
MeTLP13	LOC_Os01g64700.2	*MeTLP1*	GRMZM2G378907_T03	*MeTLP5*	Sobic.001G372000.1
MeTLP6	LOC_Os02g08310.1	*MeTLP5*	GRMZM2G378907_T03	*MeTLP7*	Sobic.001G372000.1
MeTLP9	LOC_Os02g08310.1	*MeTLP10*	GRMZM2G378907_T03	*MeTLP10*	Sobic.001G372000.1
MeTLP2	LOC_Os02g47640.1	*MeTLP3*	GRMZM5G866954_T04	*MeTLP3*	Sobic.003G368000.1
MeTLP7	LOC_Os03g22655.1	*MeTLP13*	GRMZM5G866954_T04	*MeTLP13*	Sobic.003G368000.1
MeTLP1	LOC_Os03g22800.1			*MeTLP6*	Sobic.004G064700.1
MeTLP5	LOC_Os03g22800.1			*MeTLP9*	Sobic.004G064700.1
MeTLP10	LOC_Os03g22800.1			*MeTLP2*	Sobic.004G267600.1

MeTLP, cassava tubby-like gene; *TLP*, tubby-like protein gene

Table S6 The potential *cis*-acting elements in the promoter region of cassava *MeTLP*s

Classification	Name	Function	*MeTLP*
**Development-related elements**	CAT-box	cis-acting regulatory element related to meristem expression	*MeTLP13*
**Development-related elements**	GCN4_motif	cis-regulatory element involved in endosperm expression	*MeTLP3*、*MeTLP10*
**Development-related elements**	O2-site	cis-acting regulatory element involved in zein metabolism regulation	*MeTLP9*、*MeTLP11*
**Development-related elements**	RY-element	cis-acting regulatory element involved in seed-specific regulation	*MeTLP6*
**Environmental stress-related elements**	ARE	cis-acting regulatory element essential for the anaerobic induction	*MeTLP1*、*MeTLP2*、*MeTLP3*、*MeTLP4*、*MeTLP5*、*MeTLP6*、*MeTLP8*、*MeTLP9*、*MeTLP10*、*MeTLP11*、*MeTLP13*
**Environmental stress-related elements**	GC-motif	enhancer-like element involved in anoxic specific inducibility	*MeTLP8*
**Environmental stress-related elements**	LTR	cis-acting element involved in low-temperature responsiveness	*MeTLP1*、*MeTLP2*、*MeTLP4*、*MeTLP9*、*MeTLP11*、*MeTLP13*
**Environmental stress-related elements**	MBS	MYB binding site involved in drought-inducibility	*MeTLP3*、*MeTLP4*、*MeTLP11*、*MeTLP13*
**Environmental stress-related elements**	TC-rich repeats	cis-acting element involved in defense and stress responsiveness	*MeTLP5*、*MeTLP7*、*MeTLP8*、*MeTLP9*、*MeTLP11*
**Environmental stress-related elements**	WUN-motif	wound-responsive element	*MeTLP2*、*MeTLP3*、*MeTLP4*、*MeTLP7*、*MeTLP10*、*MeTLP11*
**Hormone-responsive elements**	ABRE	cis-acting element involved in the abscisic acid responsiveness	*MeTLP1*、*MeTLP3*、*MeTLP4*、*MeTLP5*、*MeTLP6*、*MeTLP8*、*MeTLP10*、*MeTLP11*
**Hormone-responsive elements**	AuxRR-core	cis-acting regulatory element involved in auxin responsiveness	*MeTLP2*、*MeTLP3*、*MeTLP9*、*MeTLP13*
**Hormone-responsive elements**	CGTCA-motif	cis-acting regulatory element involved in the MeJA-responsive	*MeTLP1*、*MeTLP2*、*MeTLP6*、*MeTLP10*、*MeTLP11*
**Hormone-responsive elements**	ERE	ethylene-responsive element	*MeTLP2*、*MeTLP4*、*MeTLP5*、*MeTLP6*、*MeTLP7*、*MeTLP9*、*MeTLP10*、*MeTLP11*、*MeTLP13*
**Hormone-responsive elements**	GARE-motif	gibberellin-responsive element	*MeTLP2*、*MeTLP8*
**Hormone-responsive elements**	P-box	gibberellin-responsive element	*MeTLP1*、*MeTLP2*、*MeTLP7*、*MeTLP8*、*MeTLP10*
**Hormone-responsive elements**	TATC-box	cis-acting element involved in gibberellin-responsiveness	*MeTLP7*、*MeTLP11*
**Hormone-responsive elements**	TCA-element	cis-acting element involved in salicylic acid responsiveness	*MeTLP1*、*MeTLP5*、*MeTLP6*
**Hormone-responsive elements**	TGACG-motif	cis-acting regulatory element involved in the MeJA-responsiveness	*MeTLP1*、*MeTLP2*、*MeTLP6*、*MeTLP10*、*MeTLP11*
**Hormone-responsive elements**	TGA-element	auxin-responsive element	*MeTLP2*、*MeTLP10*
**Light-responsive elements**	3-AF1 binding site	light responsive element	*MeTLP5*
**Light-responsive elements**	AE-box	part of a module for light response	*MeTLP1*、*MeTLP3*、*MeTLP5*、*MeTLP13*
**Light-responsive elements**	AT1-motif	part of a light responsive module	*MeTLP6*、*MeTLP9*、*MeTLP11*
**Light-responsive elements**	ATCT-motif	part of a conserved DNA module involved in light responsiveness	*MeTLP7*
**Light-responsive elements**	Box 4	part of a conserved DNA module involved in light responsiveness	*MeTLP1*、*MeTLP2*、*MeTLP3*、*MeTLP4*、*MeTLP5*、*MeTLP6*、*MeTLP7*、*MeTLP8*、*MeTLP9*、*MeTLP10*、*MeTLP11*
**Light-responsive elements**	GA-motif	part of a light responsive element	*MeTLP2*
**Light-responsive elements**	Gap-box	part of a light responsive element	*MeTLP8*、*MeTLP13*
**Light-responsive elements**	GATA-motif	part of a light responsive element	*MeTLP1*、*MeTLP10*
**Light-responsive elements**	G-box	cis-acting regulatory element involved in light responsiveness	*MeTLP1*、*MeTLP3*、*MeTLP4*、*MeTLP5*、*MeTLP6*、*MeTLP8*、*MeTLP9*、*MeTLP10*、*MeTLP11*
**Light-responsive elements**	GT1-motif	light responsive element	*MeTLP1*、*MeTLP4*、*MeTLP5*、*MeTLP9*、*MeTLP10*、*MeTLP13*
**Light-responsive elements**	I-box	part of a light responsive element	*MeTLP1*、*MeTLP2*、*MeTLP3*、*MeTLP7*、*MeTLP10*、*MeTLP13*
**Light-responsive elements**	LAMP-element	part of a light responsive element	*MeTLP1*、*MeTLP3*、*MeTLP7*、*MeTLP9*、*MeTLP12*、*MeTLP13*
**Light-responsive elements**	MRE	MYB binding site involved in light responsiveness	*MeTLP2*、*MeTLP5*
**Light-responsive elements**	Sp1	light responsive element	*MeTLP10*
**Light-responsive elements**	TCCC-motif	part of a light responsive element	*MeTLP1*、*MeTLP3*、*MeTLP5*、*MeTLP7*、*MeTLP9*、*MeTLP10*
**Light-responsive elements**	TCT-motif	part of a light responsive element	*MeTLP1*、*MeTLP6*、*MeTLP7*、*MeTLP8*、*MeTLP11*
**Other elements**	AAGAA-motif	unknown	*MeTLP1*、*MeTLP3*、*MeTLP5*、*MeTLP6*、*MeTLP9*、*MeTLP10*、*MeTLP13*

**Other elements**	ABRE3a	unknown	*MeTLP11*
**Other elements**	ABRE4	unknown	*MeTLP11*
**Other elements**	AC-II	unknown	*MeTLP8*
**Other elements**	as-1	unknown	*MeTLP1*、*MeTLP2*、*MeTLP6*、*MeTLP10*、*MeTLP11*
**Other elements**	CAG-motif	unknown	*MeTLP5*
**Other elements**	CARE	unknown	*MeTLP8*
**Other elements**	CCAAT-box	unknown	*MeTLP2*、*MeTLP8*
**Other elements**	CTAG-motif	unknown	*MeTLP3*
**Other elements**	dOCT	unknown	*MeTLP8*
**Other elements**	DRE core	unknown	*MeTLP2*
**Other elements**	DRE1	unknown	*MeTLP8*、*MeTLP11*
**Other elements**	F-box	unknown	*MeTLP5*
**Other elements**	GTGGC-motif	unknown	*MeTLP8*
**Other elements**	MYB	unknown	*MeTLP1*、*MeTLP2*、*MeTLP3*、*MeTLP4*、*MeTLP5*、*MeTLP6*、*MeTLP7*、*MeTLP8*、*MeTLP9*、*MeTLP10*、*MeTLP11*、*MeTLP13*
**Other elements**	MYB recognition site	unknown	*MeTLP2*、*MeTLP8*
**Other elements**	Myb-binding site	unknown	*MeTLP2*、*MeTLP5*、*MeTLP8*、*MeTLP9*
**Other elements**	MYB-like sequence	unknown	*MeTLP1*、*MeTLP3*、*MeTLP13*
**Other elements**	MYC	unknown	*MeTLP1*、*MeTLP2*、*MeTLP3*、*MeTLP4*、*MeTLP5*、*MeTLP6*、*MeTLP7*、*MeTLP8*、*MeTLP10*、*MeTLP11*、*MeTLP13*
**Other elements**	STRE	unknown	*MeTLP1*、*MeTLP2*、*MeTLP3*、*MeTLP4*、*MeTLP5*、*MeTLP6*、*MeTLP7*、*MeTLP10*、*MeTLP11*、*MeTLP13*
**Other elements**	TATA	unknown	*MeTLP2*、*MeTLP4*、*MeTLP7*、*MeTLP9*、*MeTLP11*
**Other elements**	TCA	unknown	*MeTLP1*、*MeTLP6*、*MeTLP8*、*MeTLP9*、*MeTLP10*
**Other elements**	Unnamed__1	unknown	*MeTLP2*、*MeTLP3*、*MeTLP4*、*MeTLP5*、*MeTLP6*、*MeTLP8*、*MeTLP9*、*MeTLP10*
**Other elements**	Unnamed__2	unknown	*MeTLP3*、*MeTLP8*、*MeTLP9*、*MeTLP13*
**Other elements**	Unnamed__4	unknown	*MeTLP1*、*MeTLP2*、*MeTLP3*、*MeTLP4*、*MeTLP5*、*MeTLP6*、*MeTLP7*、*MeTLP8*、*MeTLP9*、*MeTLP10*、*MeTLP11*、*MeTLP13*
**Other elements**	Unnamed__6	unknown	*MeTLP4*、*MeTLP7*、*MeTLP10*
**Other elements**	W box	unknown	*MeTLP1*、*MeTLP4*、*MeTLP5*、*MeTLP7*、*MeTLP10*、*MeTLP11*、*MeTLP13*
**Other elements**	WRE3	unknown	*MeTLP1*、*MeTLP2*、*MeTLP4*、*MeTLP7*、*MeTLP8*、*MeTLP10*、*MeTLP11*
**Promoter-related elements**	CAAT-box	common cis-acting element in promoter and enhancer regions	*MeTLP1*、*MeTLP2*、*MeTLP3*、*MeTLP4*、*MeTLP5*、*MeTLP6*、*MeTLP7*、*MeTLP8*、*MeTLP9*、*MeTLP10*、*MeTLP11*、*MeTLP12*、*MeTLP13*
**Promoter-related elements**	TATA-box	core promoter element around -30 of transcription start	*MeTLP1*、*MeTLP2*、*MeTLP3*、*MeTLP4*、*MeTLP5*、*MeTLP6*、*MeTLP7*、*MeTLP8*、*MeTLP9*、*MeTLP10*、*MeTLP11*、*MeTLP12*、*MeTLP13*
**Site-binding related elements**	AT-rich element	binding site of AT-rich DNA binding protein (ATBP-1)	*MeTLP10*、*MeTLP11*、*MeTLP13*
**Site-binding related elements**	MBSI	MYB binding site involved in flavonoid biosynthetic genes regulation	*MeTLP11*

MeTLP, cassava tubby-like gene

Table S7 The expression profiles of cassava *MeTLP*s in 11 tissues of 3-month-old TME 204 cassava plants based on the GEO database submitted by Wilson et al. (2017)

MeTLP	Tissue										
	**Friable embryogenic calli**	**Fibrous roots**	**Lateral buds**	**Leaves**	**Mid veins**	**Somatic organized embryogenic structures**	**Petioles**	**Root apical meristems**	**Shoot apical meristems**	**Stems**	**Storage roots**
MeTLP1	29.61	38.98	39.64	37.92	36.66	52.28	37.71	27.38	33.64	38.41	37.67
MeTLP2	28.10	13.06	30.51	15.50	17.54	47.82	24.57	22.00	34.58	20.96	33.33
MeTLP3	37.36	61.91	36.36	25.78	29.83	40.71	38.25	21.70	32.10	36.32	36.96
MeTLP4	27.09	21.06	30.45	26.62	26.58	27.33	24.19	14.20	27.94	21.00	31.69
MeTLP5	10.24	11.37	17.98	39.58	33.42	10.57	33.02	4.27	11.31	23.15	26.35
MeTLP6	44.55	3.42	9.08	3.50	6.25	29.45	15.12	24.80	22.32	8.78	0.79
MeTLP7	20.46	20.04	36.84	51.13	41.40	37.87	29.36	30.50	42.20	24.22	12.60
MeTLP8	11.60	8.22	11.54	21.98	20.20	31.24	19.67	9.67	16.95	27.78	26.56
MeTLP9	12.74	0.82	4.34	0.16	1.01	2.09	1.54	3.34	10.92	1.26	0.18
MeTLP10	13.73	24.05	21.70	37.11	35.99	17.33	28.39	3.40	13.18	58.85	86.73
MeTLP11	20.98	23.68	22.44	21.80	21.30	14.86	19.84	11.64	15.33	19.34	24.34
MeTLP12	46.11	17.46	28.77	9.70	22.00	96.38	67.35	15.36	19.30	82.77	83.98
MeTLP13	47.18	33.85	41.31	39.74	59.56	51.51	44.69	27.23	38.40	58.76	32.75

GEO, Gene Expression Omnibus; *MeTLP*, cassava tubby-like gene

Wilson MC, Mutka AM, Hummel AW, Berry J, Chauhan RD, Vijayaraghavan A, Taylor NJ, Voytas DF, Chitwood DH, Bart RS (2017) Gene expression atlas for the food security crop cassava. New Phytol 213:1632-1641.
