# Supplementary material for: Decrypting tubby-like protein gene family of multiple functions in starch root crop cassava
Source: AoB Plants. 2019 Nov 25;11(6):plz075. doi: 10.1093/aobpla/plz075 (PMC6920310; doi:10.1093/aobpla/plz075)
Supplement: plz075_suppl_Supplementary_Table_S1 [file plz075_suppl_supplementary_table_s1.docx]

| Table S1 The accession number of the public high-throughput RNA-seq read archives databases submitted by Hu et al (2016b) | |
| --- | --- |
| VarietyˍTissue | **Accession number** |
| W14_middle storage root | SRR1298996 |
| W14_leaf | SRR1298998 |
| W14_stem | SRR1298999 |
| Arg7_middle storage root | SRR1299006 |
| Arg7_leaf | SRR1299009 |
| Arg7_stem | SRR1299008 |
| KU50_root ear storage root | SRR1299001 |
| KU50_middle storage root | SRR1299002 |
| KU50_last storage root | SRR1299003 |
| Arg7_early storage root | SRR1299005 |
| Arg7_middle storage root | SRR1299006 |
| Arg7_last storage root | SRR1299007 |
| Not: Hu W, Yang HB, Yan Y, Wei YX, Tie WW, Ding ZH, Zuo J, Peng M, Li KM (2016b) Genome-wide characterization and analysis of bZIP transcription factor gene family related to abiotic stress in cassava. Sci Rep 6:22783. W14, cassava wild species. | |
