# Supplementary material for: Decrypting tubby-like protein gene family of multiple functions in starch root crop cassava
Source: AoB Plants. 2019 Nov 25;11(6):plz075. doi: 10.1093/aobpla/plz075 (PMC6920310; doi:10.1093/aobpla/plz075)
Supplement: plz075_suppl_Supplementary_Table_S8 [file plz075_suppl_supplementary_table_s8.docx]

| Table S8 The expression of cassava *MeTLP*s in different tissues of cultivars Ku50 and Arg7, and wild species W14 based on the high-throughput SRA databases submitted by Hu et al (2016b) | | | | | | | | | | | | | |
| --- | --- | --- | --- | --- | --- | --- | --- | --- | --- | --- | --- | --- | --- |
| *MeTLP* | **Varity/Species** | | | | | | | | | | | |  |
|  | **Arg7** | | | | |  | **KU50** | | |  | **Wild species W14** | | |
|  | **Early**  **Storage**  **roots** | **Middle storage roots** | **Last storage roots** | **Stems** | **Leaves** |  | **Early**  **Storage**  **roots** | **Middle storage roots** | **Last storage roots** |  | **Middle storage roots** | **Stems** | **Leaves** |
| *MeTLP1* | 15.59 | 17.05 | 17.49 | 27.62 | 17.58 |  | 16.67 | 13.91 | 18.70 |  | 14.79 | 39.95 | 22.93 |
| *MeTLP2* | 28.06 | 19.54 | 25.95 | 16.02 | 9.42 |  | 37.68 | 36.58 | 37.34 |  | 20.93 | 20.12 | 12.97 |
| *MeTLP3* | 27.96 | 21.67 | 25.28 | 25.84 | 22.64 |  | 27.90 | 24.77 | 22.64 |  | 21.77 | 23.80 | 22.04 |
| *MeTLP4* | 9.51 | 12.73 | 14.90 | 14.79 | 11.40 |  | 13.83 | 14.99 | 13.66 |  | 8.23 | 19.11 | 16.53 |
| *MeTLP5* | 5.74 | 3.96 | 3.09 | 7.65 | 3.30 |  | 6.06 | 7.01 | 9.65 |  | 4.22 | 5.96 | 9.02 |
| *MeTLP6* | 5.49 | 2.96 | 1.22 | 3.11 | 0.48 |  | 3.45 | 4.46 | 3.55 |  | 6.40 | 4.40 | 0.55 |
| *MeTLP7* | 14.62 | 11.83 | 12.71 | 31.18 | 15.35 |  | 18.59 | 14.94 | 17.99 |  | 16.21 | 39.04 | 25.61 |
| *MeTLP8* | 5.00 | 3.14 | 2.33 | 27.06 | 18.44 |  | 7.23 | 3.80 | 8.80 |  | 5.53 | 19.04 | 4.53 |
| *MeTLP9* | 0.34 | 0.19 | 0.08 | 0.53 | 0.10 |  | 0.23 | 0.30 | 0.37 |  | 0.00 | 0.18 | 0.00 |
| *MeTLP10* | 7.12 | 1.13 | 1.58 | 7.53 | 1.74 |  | 9.15 | 7.29 | 8.85 |  | 19.69 | 18.63 | 20.48 |
| *MeTLP11* | 20.92 | 22.01 | 21.16 | 10.81 | 12.89 |  | 21.77 | 24.99 | 20.97 |  | 17.72 | 14.67 | 15.57 |
| *MeTLP12* | 25.75 | 8.26 | 13.66 | 42.82 | 9.40 |  | 87.08 | 49.68 | 72.67 |  | 58.17 | 35.01 | 11.02 |
| *MeTLP13* | 66.44 | 167.83 | 129.93 | 65.58 | 58.23 |  | 39.05 | 78.72 | 51.43 |  | 58.19 | 42.69 | 71.92 |
| *MeTLP*, cassava tubby-like gene  Not: Hu W, Yang HB, Yan Y, Wei YX, Tie WW, Ding ZH, Zuo J, Peng M, Li KM (2016b) Genome-wide characterization and analysis of bZIP transcription factor gene family related to abiotic stress in cassava. Sci Rep 6:22783. | | | | | | | | | | | | | |
